# Electro-Myo-Stimulation Induced Tic Exacerbation – Increased Tendencies for the Formation of Perception-Action Links in Tourette Syndrome

**DOI:** 10.5334/tohm.547

**Published:** 2020-10-08

**Authors:** Anne Weissbach, Maximilian Kleimaker, Tobias Bäumer, Christian Beste, Alexander Münchau

**Affiliations:** 1 Institute of Systems Motor Science, University of Lübeck, Lübeck, DE; 2 Institute of Neurogenetics, University of Lübeck, Lübeck, DE; 3 Cognitive Neurophysiology, Department of Child and Adolescent Psychiatry, Faculty of Medicine of the TU Dresden, Dresden, DE

**Keywords:** Gilles de la Tourette syndrome (GTS), electro-myo-stimulation (EMS), tic exacerbation, perception-action processing

## Abstract

**Background:**

Gilles de la Tourette syndrome (GTS) is a neuropsychiatric disorder defined by motor and phonic tics. Sensory stimuli can trigger tics, which suggests that GTS is a disorder of perception-action processing rather than a pure motor disorder.

**Case report:**

We describe a GTS patient that developed exacerbation of tics after transcutaneous electro-myo-stimulation (YGTSS pre-EMS 27/100, post-EMS 69/100).

**Discussion:**

If behaviorally irrelevant stimuli exacerbate tics, there might be a high readiness of the motor system to respond to any stimulus in these patients. In addition to tighter binding between previously established perception-action links, the likelihood for the formation of automatic perception-action links might also be higher in GTS.

## Case report

Gilles de la Tourette syndrome (GTS) is a neuropsychiatric disorder defined by motor and phonic tics [1]. Tics are rapid movements or sounds, resembling spontaneous movement/sound fragments, but appear misplaced in time and context. In most patients, there are also unpleasant sensations preceding tics, typically described as urges. Therefore, tics might be interpreted as a reaction to certain bodily sensations [2]. Patients with GTS have also been described to be particularly sensitive to external stimuli [3], which is not due to increased perception of low-level stimuli, as basic perception thresholds are normal in GTS [4]. Moreover, sensory stimuli can trigger tics, which along with premonitory urges suggests that GTS is a disorder of perception-action processing rather than a pure motor disorder [5]. Whereas premonitory urges are acknowledged as an integral feature of GTS, hypersensitivity to external stimuli and its untoward consequences are less well known.

Here, we describe a 28-years-old patient with GTS presenting to our GTS clinic because of considerable exacerbation of motor tics after commencing transcutaneous electro-myo-stimulation (EMS) for sportive purposes using an EMS jacket. EMS is a technique where participants wear electrodes that apply a low electric current (mean 60 mA, 69 Hz, 266 μs) [6] to the muscles during isometric contraction to induce muscle fiber hypertrophy. EMS is widely used in athletes, but also patients who cannot perform voluntary exercise, to increase muscle mass, strength, and exercise capacity [6]. EMS training, as used by our patient, is applied through guidelines and regulations [7], and the typical sensory experience associated with EMS in the majority of healthy individuals is not unpleasant, but a light muscle activation.

Similar to classical EMS training [6], our patient applied alternating turns of four seconds stimulation and four seconds rest for 15 minutes three times per week. His stimulation parameters were similar to the above mention standard parameters and were kept unchanged during all training sessions. After the first training session, he noted a slight increase of tics. After the next two training sessions, within the same week, he realized an even further increase of motor and vocal tic severity with high frequent rapid head jerks, shoulder elevations, trunk twists, and arm jerks, as well as clearing his throat and puffing (YGTSS global severity score pre-EMS 27/100, post-EMS 69/100). His urge to carry out these tics also increased considerably. Symptom exacerbations always occurred immediately after each EMS applications and lasted for four hours. The days following EMS, tics and urge severity had returned to the level he was used to for many years, which is why he stopped EMS training. The patient reported no stress or discomfort during the training. After an interval of several weeks, he re-tried EMS, which again led to an increase of tic severity as described above. Our patient had not exhibited somatic hypersensitivity with respect to other sensory modalities (visual, auditory, olfactory stimuli). He did not report any abnormalities in his sense of smell, vision and hearing. His neurological examination was, beside his tic disorder, completely normal including sensory examination of touch, pain, proprioception, and the sense of vibration and pressure.

This observation of low current-induced tic exacerbation underscores the tight link between sudden alterations in bodily perception and tic severity in GTS, which could be interpreted such that sensitivity to exteroceptive stimuli is increased in GTS. However, this is but one side of the coin. If behaviorally irrelevant stimuli (low current) readily trigger or exacerbate tics, then this also implies that in GTS, there is high readiness of the motor system to respond to any sensory stimulus. That perception-action binding might indeed be stronger in GTS has been proposed [5] and also shown experimentally [8]. This case suggests that in addition to tighter binding between previously established perception-action links, there is also a higher likelihood of the formation of automatic perception-action links, the neural basis of which warrants further neurophysiological studies.

However, there are certain limitations to our case report. It is, to the best of our knowledge, so far the first and only description of EMS-induced increase of tic severity in a GTS patient. It is, therefore, not possible to draw direct and general conclusions to the whole population of GTS patients. Moreover, we did not include a placebo control in our case report. Therefore, we cannot state with certainty that the tic exacerbation is related to the EMS training only. Other factors including stress, anxiety, fatigue, and sensory hyperexcitability might also have contributed to the enhancement of symptoms. Further potential clinical trials could use a placebo stimulation to validate untoward EMS effects in closer detail. Regarding neurophysiological studies, further investigations of the role of the peripheral sensory system in perception-action binding are needed.
